# The long non-coding RNA ERIC is regulated by E2F and modulates the cellular response to DNA damage

**DOI:** 10.1186/1476-4598-12-131

**Published:** 2013-10-29

**Authors:** Orit Feldstein, Tal Nizri, Tirza Doniger, Jasmine Jacob, Gideon Rechavi, Doron Ginsberg

**Affiliations:** 1 The Mina and Everard Goodman Faculty of Life Science, Bar Ilan University, Ramat Gan 52900, Israel; 2 Cancer Research Center, Chaim Sheba Medical Center, Tel Hashomer, Israel; 3 Sackler School of Medicine, Tel Aviv University, Tel Aviv, Israel

**Keywords:** E2F1, Long non-coding RNA, Apoptosis

## Abstract

**Background:**

The human genome encodes thousands of unique long non-coding RNAs (lncRNAs), and these transcripts are emerging as critical regulators of gene expression and cell fate. However, the transcriptional regulation of their expression is not fully understood. The pivotal transcription factor E2F1 which can induce both proliferation and cell death, is a critical downstream target of the tumor suppressor, RB. The retinoblastoma pathway is often inactivated in human tumors resulting in deregulated E2F activity.

**Results:**

Here, we report that lncRNA XLOC 006942, which we named ERIC, is regulated by E2F1 and, most probably, also E2F3. We show that expression levels of ERIC were elevated upon activation of exogenous E2F1, E2F3 or endogenous E2Fs. Moreover, knockdown of either E2F1 or E2F3 reduced ERIC levels and endogenous E2F1 binds ERIC’s promoter. Expression of ERIC was cell cycle regulated and peaked in G1 in an E2F1-dependent manner. Inhibition of ERIC expression increased E2F1-mediated apoptosis, suggesting that E2F1 and ERIC constitute a negative feedback loop that modulates E2F1 activity. Furthermore, ERIC levels were increased following DNA damage by the chemotherapeutic drug Etoposide, and inhibition of ERIC expression enhanced Etoposide -induced apoptosis.

**Conclusions:**

Our data identify ERIC as a novel lncRNA that is transcriptionally regulated by E2Fs, and restricts apoptosis induced by E2F1, as well as by DNA damage.

## Background

Genome-wide transcriptome studies have revealed that the mammalian genome encodes a novel class of regulatory genes encoding long non-coding RNAs (lncRNAs), which are >200 bases in length but lack significant open reading frames [1]. It is believed that the genome encodes at least as many lncRNAs as known protein-coding genes [1]. The expression of many lncRNAs is tissue specific and, in some cases, restricted to particular developmental contexts [1,2]. Furthermore, thousands of lncRNAs were found to be evolutionarily conserved [3,4] and exhibit expression patterns that correlate with various cellular processes [3-9]. It is now considered likely that this class of ncRNA represents a significant feature of normal cellular networks. Specifically, increasing evidence suggests that lncRNAs play a critical role in regulation of diverse cellular processes such as stem cell pluripotency, development, cell growth, and apoptosis [3-9]. Given their abundance and regulatory potential, it is likely that some lncRNAs are involved in tumor initiation and progression. In support of this notion, several lncRNAs are frequently aberrantly expressed in various human cancers, with potential roles in both oncogenic and tumor suppressive pathways [10-14]. Furthermore, lncRNAs were shown to play active roles in modulating the cancer epigenome [15].

Recent studies have suggested a number of modes of action for lncRNAs [16], most notably the regulation of epigenetic marks and gene expression [6,17-19]. Also, lncRNAs were shown to function as decoy, scaffold or guide molecules [1]. Some lncRNAs act in cis to regulate transcription of a nearby gene(s) [20,21], while other lncRNAs can act in trans to repress transcription [22].

Many, although not all, lncRNAs are generated and processed through mechanisms similar to those that process mRNA. Specifically, many lncRNAs are transcribed by RNA polymerase II [23], spliced and polyadenylated [24]. Additionally, lncRNA promoters are generally bound and regulated by transcription factors known to influence mRNA transcription [3,25,26]. One of the p53-regulated lncRNAs, lncRNA-p21 was shown to play a pivotal role in the p53-dependent apoptotic response to DNA damage [22]. Another lncRNA named PANDA was found to play a critical role in inhibiting p53-mediated apoptosis [5].

E2Fs are transcription factors best known for their involvement in the timely regulation of gene expression required for cell cycle progression [27]. Members of the E2F family are downstream effectors of the tumor suppressor, pRB. The critical role of the RB/E2F pathway in xnormal cellular proliferation is highlighted by the common incidence among human tumors of pathway mutations that result in deregulated E2F activity [28]. This deregulated E2F activity results in uncontrolled cell proliferation, a hallmark of tumor cells.

In addition to acting as fundamental regulators of proliferation, E2Fs modulate diverse cellular functions, such as DNA repair, differentiation and development [29,30]. At least one member of the E2F family, namely E2F1, can also trigger apoptosis [27,31] and autophagy [32-34]. E2F1-induced apoptosis is mediated by both p53-dependent and p53-independent pathways [27,35]. In line with the effects of E2Fs on both proliferation and apoptosis, E2F1 functions *in vivo* in a context-dependent manner as an oncogene or a tumor suppressor [36]. Currently, little is known about transcriptional regulation of lncRNAs by E2F1 or the role played by lncRNAs in E2F1-regulated biological functions.

In the present study, we characterized XLOC 006942, a novel lncRNA that we named ERIC (E2F1-Regulated Inhibitor of Cell death). We demonstrate here that ERIC is transcriptionally up-regulated by E2F1 as well as by DNA damage, and we show that inhibition of ERIC augments apoptotic cell death induced by either E2F1 or a DNA damaging agent.

## Results

### E2F directly regulates expression of ERIC in a p53-independent manner

**Figure 1:**
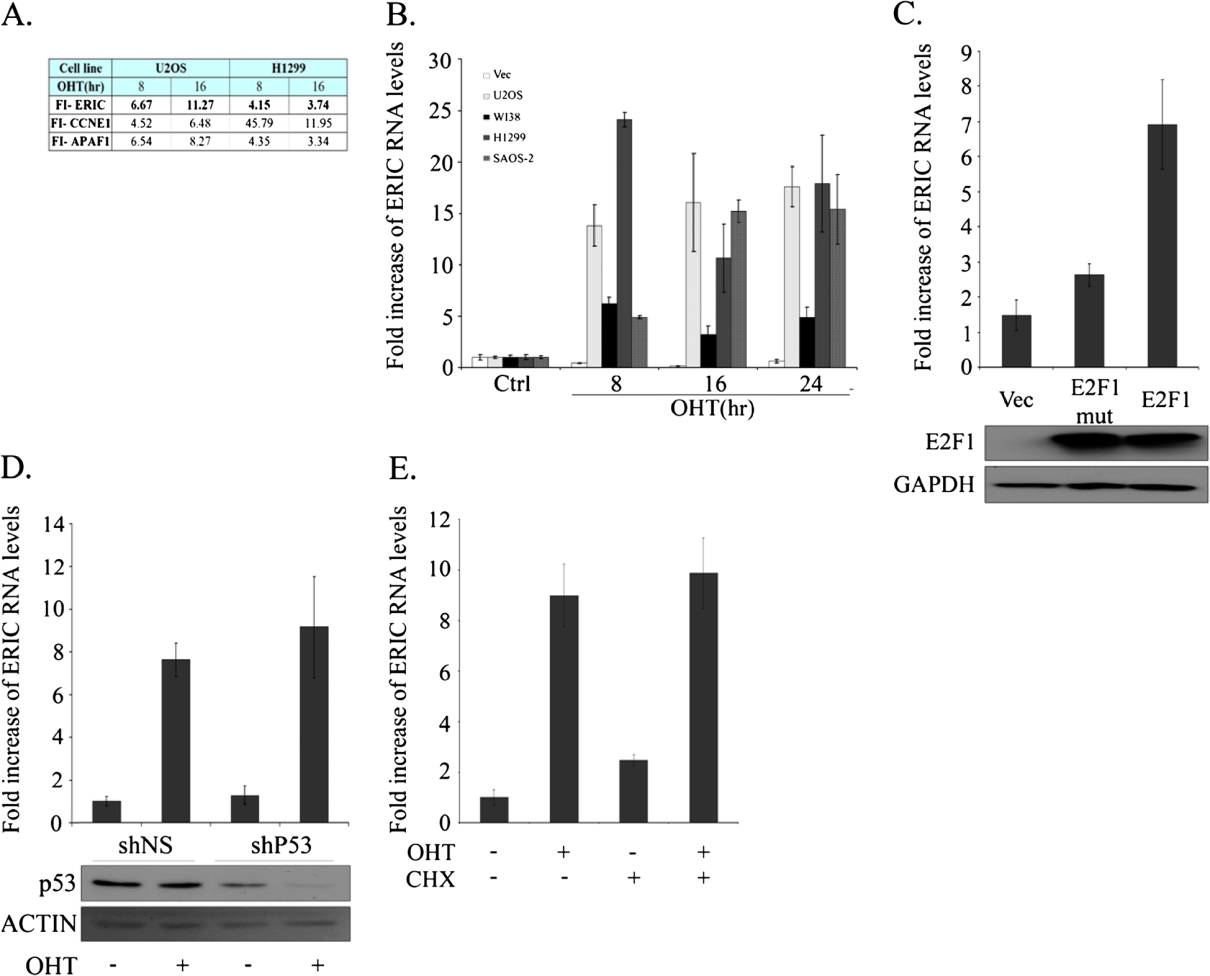
**Ectopic E2F1 expression directly upregulates ERIC RNA levels. A)** U2OS and H1299 cells containing conditionally active E2F1 were induced to activate E2F1 by OHT (times indicated). RNA was extracted and RNA deep-sequencing analysis was employed. FI- fold of increase in RNA levels after E2F1 induction, determined by RNA sequencing. **B)** U2OS, H1299, WI38 and SAOS-2 cells containing conditionally active E2F1 were induced to activate E2F1 by addition of OHT (times indicated). RNA was extracted, and ERIC RNA levels determined by Real- time RT-PCR and normalized to GAPDH. U2OS cells containing an empty vector (vec) served as a control. **C)** U2OS cells were transfected with an empty vector (Vec) or a vector expressing either wild type E2F1 (E2F1) or mutant E2F1 (E2F1 mut). Upper panel- RNA was extracted and ERIC RNA levels determined by Real-time RT-PCR and normalized to GAPDH. Lower panel- Proteins were extracted and western-blot analysis performed using antibodies directed against E2F1 and GAPDH. **D)** Induction of ERIC by E2F1 is p53-independent. U2OS cells stably expressing ER- E2F1 were infected with either a retrovirus expressing non specific shRNA (shNS) or a retrovirus expressing shRNA directed against p53 (shp53). Cells were left untreated (-) or incubated with OHT (100nM) for 16 hr (+). Upper panel- RNA was extracted and ERIC RNA levels were determined by real-time RT-PCR and normalized to GAPDH. Lower panel- Proteins were extracted and western-blot analysis was performed using antibodies directed against p53 and Actin. **E)** Induction of ERIC does not require protein synthesis. U2OS cells expressing ER-E2F1 were left untreated or incubated with OHT (100 nM, 8 hr); cells were then treated or not with 10 μg/mL cycloheximide (CHX) for 8 h. RNA was extracted and ERIC RNA levels determined by real-time RT-PCR and normalized to GAPDH. The average of two independent experiments is presented.

To explore if E2F1 regulates expression of long non-coding RNAs (lncRNAs) we took advantage of a human osteosarcoma cell line U2OS and a human lung carcinoma cell line H1299 that each express conditionally active E2F1, namely ER-E2F1 [37]. We reasoned that E2F1 activation might result in increased expression of not only protein coding genes but also lncRNAs. We activated E2F1 for either 8 or 16 hours and surveyed RNA transcript levels in the U2OS and H1299 cells using RNA Seq analysis. In accord with published data, many known E2F1-regulated protein-coding genes exhibited increased expression upon E2F1 activation (Figure 1A and data not shown). The E2F1-induced upregulation of these genes validated our experimental system and served as an internal positive control. Notably, in line with our hypothesis, we detected increased expression of many lncRNAs upon activation of E2F1. Our analysis of the RNA Seq data was based on the recently published genomic coordinates of 8,196 long intergenic non-coding RNAs (lincRNAs) [38]. Out of these 8,196 lincRNAs, 5,998 lincRNAs were not expressed in either of the two cell lines we analyzed. When studying the remaining 2,198 lincRNAs that were expressed in at least one cell line we found that expression of 137 lincRNAs was upregulated more than two fold at both time points in at least one of the cell lines after activation of E2F1 (Additional file 1: Table S1). From this group of lincRNAs putatively regulated by E2F1, we chose to focus on lincRNA XLOC 006942, which we named ERIC (E2F1-Regulated Inhibitor of Cell death); this lncRNA exhibited increased RNA levels upon activation of E2F1 in both cell lines (Figure 1A). ERIC (also termed TCONS_00014875) is located on chromosome 8 (chr8:141646242-141648531) at a position corresponding to band 8q24.3 on a somatic map, and is transcribed from the plus strand. ERIC is composed of two exons and its transcript size is 1745 bp.

To validate and extend the RNA-Seq data, we performed real-time PCR analysis of ERIC levels in four cell lines, each expressing the conditionally active E2F1, ER-E2F1. As can be seen in Figure 1B, activation of the ectopic E2F1 by addition of its inducer, 4-hydroxytamoxifen (OHT), resulted in a significant increase in ERIC RNA levels in the U2OS and H1299 lines described above, as well as another human osteosarcoma cell line, SAOS-2, and WI38 human embryonic lung fibroblasts. As expected, addition of OHT to U2OS cells lacking the inducible E2F1 (“Vec”) did not affect ERIC mRNA levels, validating the role of E2F1 in the upregulation (Figure 1B). Similarly, transient transfection of wt E2F1, but not of a mutant E2F1 that does not bind DNA (E2F1E132), into U2OS cells also resulted in a significant increase in ERIC RNA levels (Figure 1C). Notably, activation of conditional E2F3 also resulted in significantly increased ERIC RNA levels (Additional file 2: Figure S1A). Taken together, these data suggest that an activity common to E2F1 and E2F3 is responsible for up-regulating ERIC expression.

In addition to directly activating gene expression, E2F1 can also activate p53 [39]; therefore activation of a gene by E2F1 may represent indirect activation via p53. However, the observed E2F1-mediated upregulation of ERIC was p53-independent since it is also observed in H1299 cells and SAOS-2 cells, both of which lack wt p53. Moreover, we found that activation of conditional E2F1 induced ERIC expression levels in the p53 proficient U2OS cell line even in the presence of shRNA directed against p53 (Figure 1D). To further examine whether E2F regulates ERIC directly or indirectly, ectopic E2F1 was activated in the presence of the inhibitor of protein synthesis, cycloheximide. In support of direct regulation, E2F1 activation in the presence of cycloheximide did not mitigate ERIC RNA induction (Figure 1E).

### Endogenous E2F regulates ERIC expression

**Figure 2:**
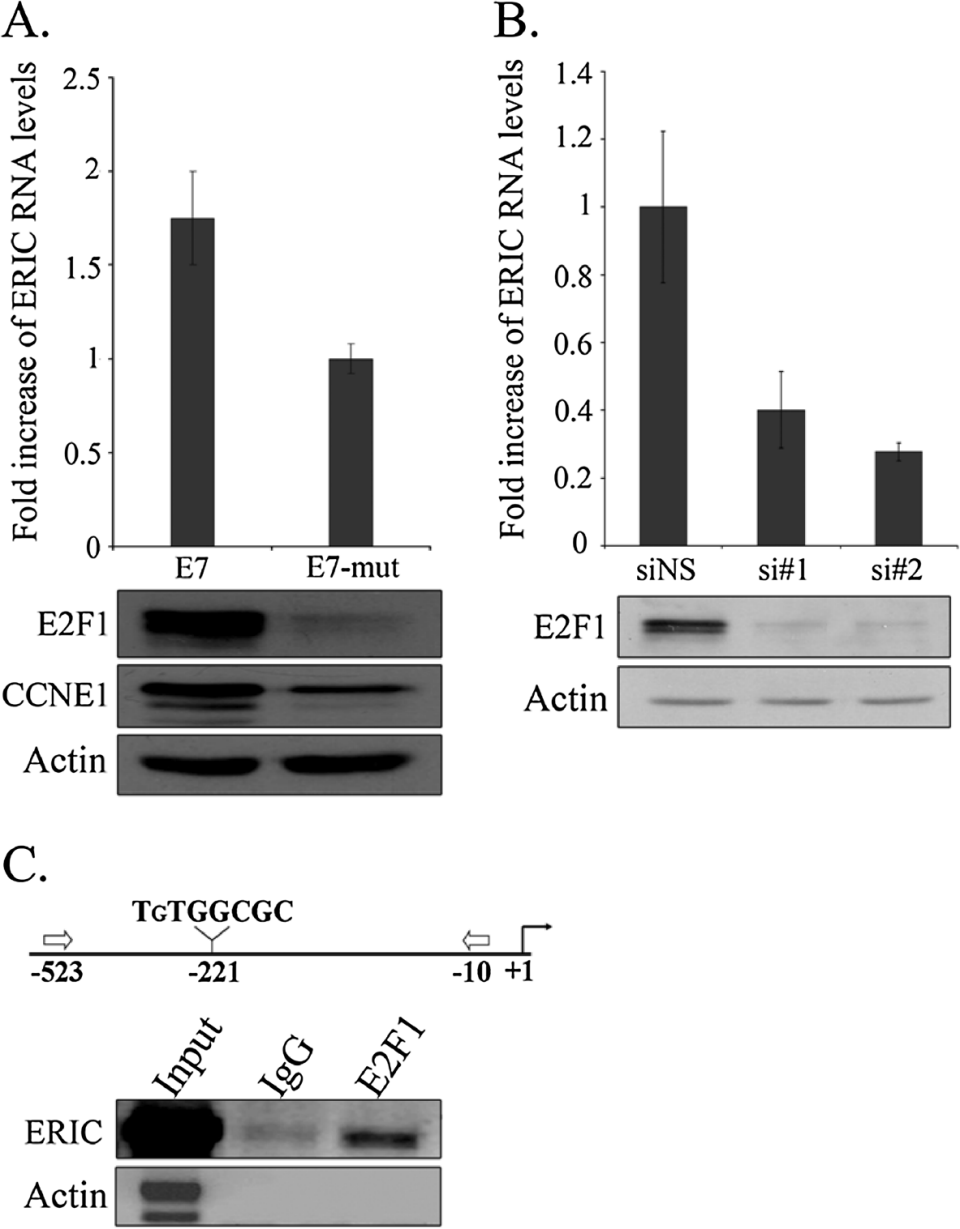
**Endogenous E2F1 regulates expression of ERIC. A)** WI38 cells were infected with a retrovirus expressing either wild-type E7 (E7) or an RB-binding–deficient E7 mutant (E7-mut). Upper panel-RNA was extracted and ERIC RNA levels determined by Real-time RT-PCR and normalized to GAPDH levels. Lower panel- proteins were extracted and western blot analysis performed using antibodies directed against E2F1, CCNE1 and Actin. **B)** Upper panel- U2OS cells were transfected with either a nonspecific siRNA (siNS) or an siRNA directed against E2F1 (si#1 and si#2). RNA was extracted and ERIC RNA levels determined by Real-time RT-PCR and normalized to GAPDH levels. Lower panel- Proteins were extracted from cells and western blot analysis performed using antibodies directed against E2F1 and actin. **C)** Upper panel- a schematic representation of the human ERIC promoter. The E2F-binding site is represented as 8-mer nucleotide sequences. The transcription start site (+1) is indicated by an arrow. The DNA fragment amplified by RT-PCR is represented by arrows (-523/-10). Lower panel- Chromatin Immuno Precipitation analysis was conducted using U2OS cells. Cross-linked chromatin was precipitated using antibodies specific to E2F1 or IgG. Then, ERIC and Actin promoter fragments were amplified by RT-PCR. Input DNA represents 0.5% of total chromatin.

To further support the notion that endogenous E2Fs are capable of regulating ERIC expression, we took advantage of the Human Papilloma Virus oncoprotein E7, which disrupts RB/E2F complexes, resulting in activation of endogenous E2Fs. In line with our results using inducible E2F, expression of E7 in WI38 human fibroblasts elevated the levels of ERIC, when compared to a mutant E7, E7Δ21-35, which does not disrupt RB/E2F complexes (Figure 2A). To further confirm the involvement of endogenous E2F1 in controlling ERIC expression, the effect of E2F1 knockdown on ERIC RNA levels was studied. To this end, two distinct siRNAs directed against E2F1 were introduced to U2OS cells; as expected, siRNA inhibition resulted in reduction of E2F1 protein levels (Figure 2B, lower panel). When the effect of knockdown on ERIC was tested, E2F1 knockdown resulted in significantly reduced endogenous ERIC RNA levels (Figure 2B). Similar results were obtained upon silencing of E2F1 in SAOS-2 cells (Additional file 3: Figure S2). Additionally, E2F3 knockdown in U2OS cells, by two distinct siRNAs, resulted in reduced endogenous ERIC RNA levels (Additional file 2: Figure S1B, C). Also, analysis of the human genomic sequence upstream to ERIC identified a putative E2F binding site at –221/-214 (TSS according to [38]) suggesting that endogenous E2Fs indeed bind upstream to ERIC. Further support for this notion was sought using Chromatin Immunoprecipitation analysis (ChIP). Chromatin was isolated from proliferating U2OS cells and incubated with an antibody directed against E2F1 and as predicted, endogenous E2F1 was detectably associated with the human genomic sequence upstream to ERIC (Figure 2C). Taken together, these data demonstrate that both endogenous and ectopically expressed E2F1 and E2F3 regulate ERIC levels.

**Figure 3:**
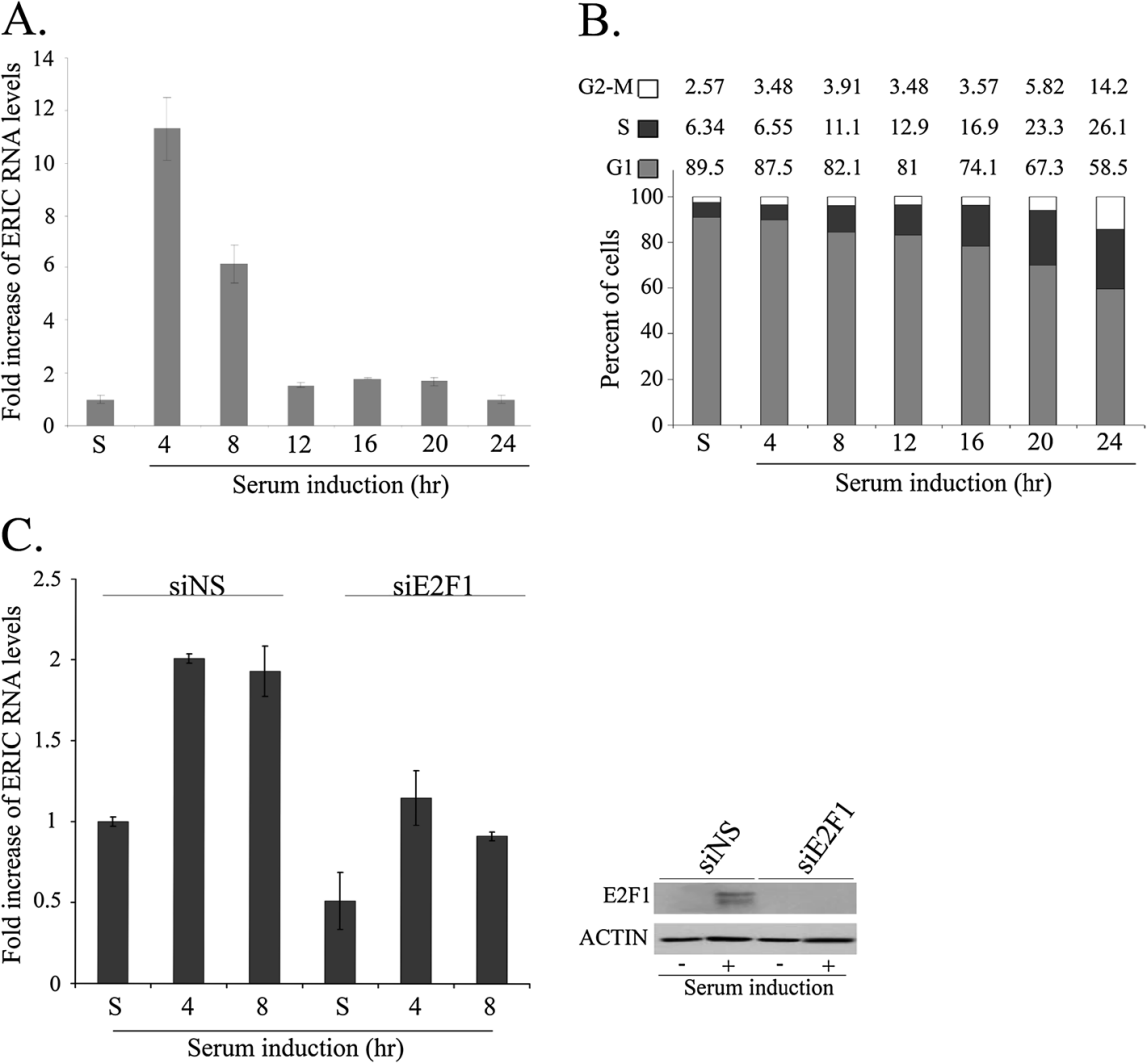
**ERIC is cell cycle regulated.** WI38 cells were growth arrested by serum deprivation (48 hours in medium with 0.1% serum, S = starvation) and then allowed to resume growth by serum addition (to a final concentration of 15%) for the times indicated. **A)** RNA was extracted and RNA levels of ERIC were determined by Real-time RT-PCR and normalized to GAPDH levels. **B)** Cell cycle distribution was determined using FACS analysis. Percentage of cells in G1, S and G2/M are indicated. **C)** WI38 cells were transfected with either a nonspecific siRNA (siNS) or an siRNA directed against E2F1 (siE2F1). Then cells were growth arrested by serum deprivation (48 hours in medium with 0.1% serum, S = starvation) and then allowed to resume growth by serum addition (to a final concentration of 15%) for the times indicated. Left panel-RNA was extracted and RNA levels of ERIC were determined by Real-time RT-PCR and normalized to GAPDH levels. Right panel-Proteins were extracted from the cells, and western blot analysis performed using antibodies directed against E2F1 and actin.

To determine whether expression of ERIC is cell cycle regulated, we examined its RNA levels in WI38 cells that were growth arrested at G1 by serum starvation and induced to reenter the cell cycle by addition of 15% FBS. Under these conditions, we detected a rapid and transient increase in ERIC RNA levels as arrested cells resumed growth (Figure 3A). Specifically, ERIC levels increased 11 and 6 fold, 4 and 8 hours after serum addition, respectively. This transient increase in ERIC levels slightly preceded G1/S transition of the serum-stimulated cells (Figure 3B). Of note, knock down of E2F1 significantly inhibits expression of ERIC both in starved cells and upon release from starvation (Figure 3C). Thus, our data suggest that endogenous E2F1 regulates ERIC expression both in cycling cells and in arrested cells that resume growth.

### ERIC inhibits E2F1-induced apoptosis

**Figure 4:**
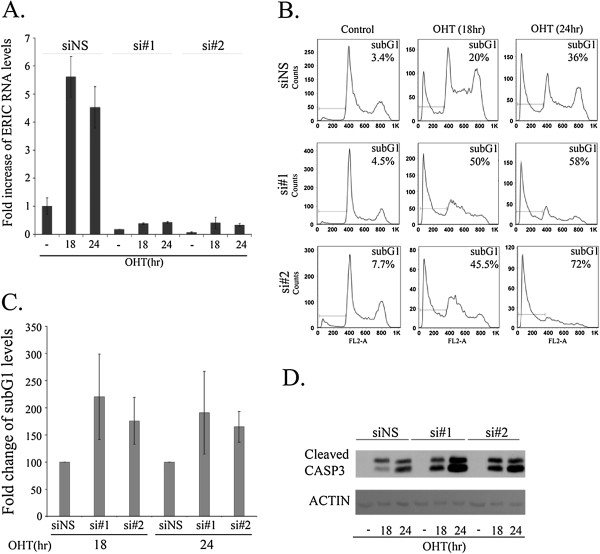
**ERIC restricts E2F1-mediated apoptosis.** U2OS cells stably expressing ER-wild type E2F1 were transfected with either a nonspecific siRNA (siNS) or an siRNA directed against ERIC (si#1 or si#2). Then, cells were left untreated or incubated with OHT (100 nM) for times indicated. **A)** RNA was extracted and ERIC RNA levels were determined by real-time RT-PCR and normalized to GAPDH levels. One representative experiment is shown out of 3 repeats. **B)** Cells were analyzed by fluorescence-activated cell sorting (FACS) analysis using propidium-iodide (PI) staining. One representative experiment is shown. Numbers represent percent of cells with a subG1 DNA content. **C)** Summary of three independent FACS experiments. **D)** Proteins were extracted from the cells, and western blot analysis performed using antibodies directed against cleaved caspase 3 and actin.

To directly determine whether ERIC plays a role in E2F1-induced biological processes, we reduced endogenous ERIC RNA levels in U2OS cells by two distinct siRNA, and examined the effect(s) on E2F1-induced G1/S transition as well as E2F1-induced apoptosis. Introduction of each of these two siRNAs resulted in significantly reduced basal levels of ERIC RNA and inhibited its up regulation by E2F1 compared to non-specific siRNA (Figure 4A). Having established the efficacy of the ERIC-specific siRNAs, we examined the effects of ERIC knockdown on E2F1-mediated biological processes. In line with previous reports, activation of ectopic E2F1 resulted in S phase entry as well as apoptosis. Apoptosis was evaluated by monitoring the percentage of cells with sub-G1 DNA content and also by monitoring the levels of cleaved caspase 3 (Figure 4B, C, D). Despite the increase of ERIC RNA levels as arrested cells resume growth (Figure 3), ablation of ERIC did not have a significant or reproducible effect on the E2F1-induced G1/S transition (Figure 4B). Similarly, ablation of ERIC did not have a significant effect on cell viability and cell cycle distribution (Figure 4B, left histograms). Interestingly, the knockdown of ERIC significantly enhanced E2F1-induced apoptosis as evident by the reproducible increase in the percentage of cells with sub G1 DNA content as well as the increase in cleaved caspase 3 (Figure 4B, C, D). This effect of ERIC ablation on E2F1-induced apoptosis was also evident in similar experiments performed using H1299 cells (Additional file 4: Figure S3) and SAOS-2 cells (data not shown). Taken together, these data suggest a negative feedback loop in which the E2F1-regulated ERIC inhibits E2F1-induced apoptosis.

### ERIC is upregulated upon DNA damage and inhibits DNA damage-induced apoptosis

**Figure 5:**
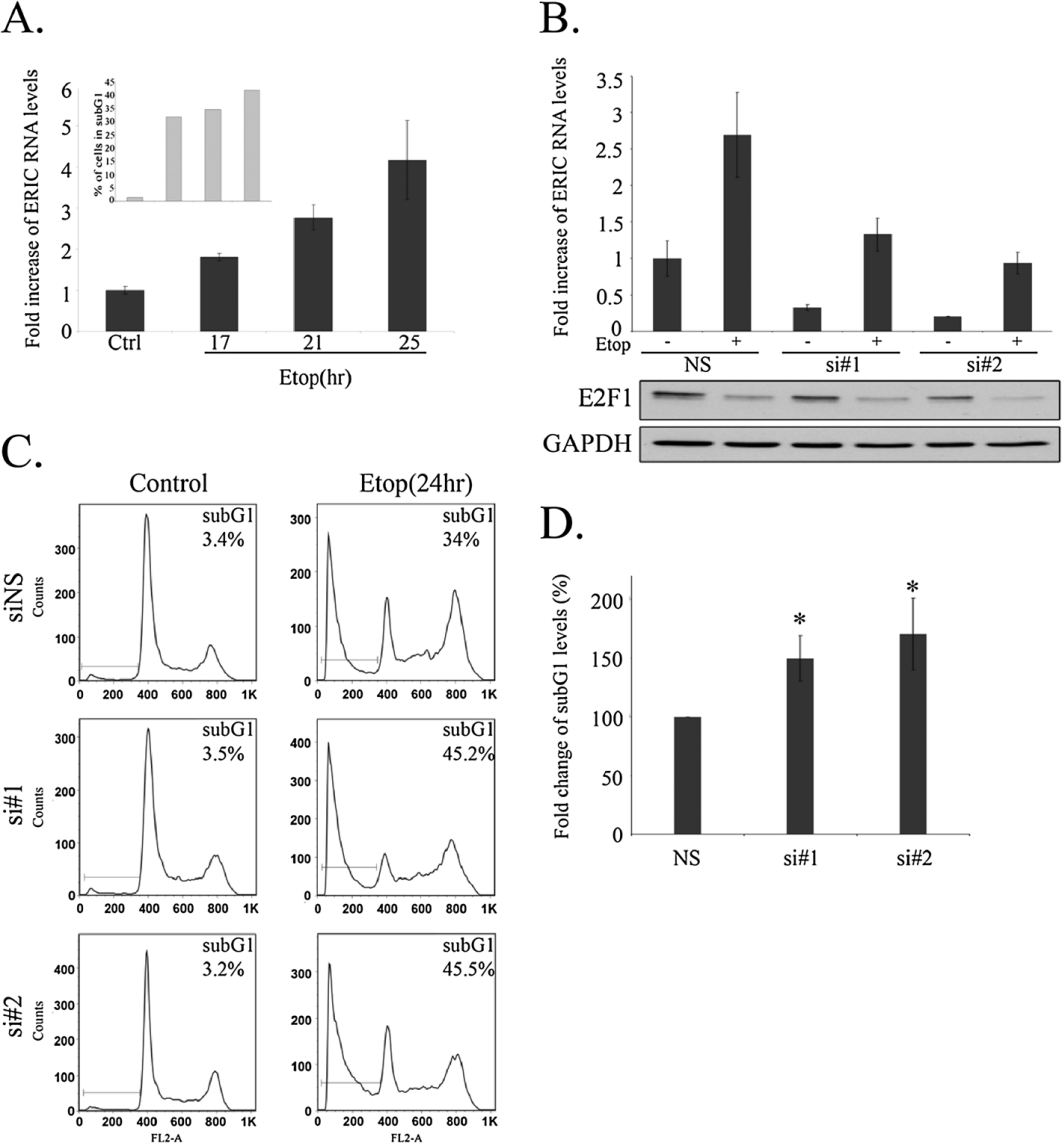
**DNA damage upregulates ERIC RNA levels, and ERIC restricts DNA damage-induced apoptosis. A)** U2OS cells were treated with etoposide (50 μg/ml) for times indicated. RNA was extracted from cells and ERIC RNA levels were determined by real-time RT-PCR and normalized to GAPDH. One representative experiment out of 4 is shown. Inner bar-graph indicates percent of cells exhibiting subG1 DNA content at each time point, as analyzed by FACS. U2OS cells were transfected with either a nonspecific siRNA (NS) or an siRNA directed against ERIC (si#1 or si#2). Then, cells were left untreated or incubated with etoposide (Etop) (50 μg/ml) for 24 hours. **B)** Upper panel- RNA was extracted and ERIC RNA levels were determined by real-time RT-PCR and normalized to GAPDH levels. Lower panel- Proteins were extracted from cells and western blot analysis performed using antibodies directed against E2F1 and GAPDH. **C)** Cells were analyzed by FACS following propidium-iodide (PI) staining. One representative experiment out of 3 is shown. Numbers represent percent of cells with a subG1 DNA content. **D)** Summarized results of three independent FACS experiments, *P value = 0.01 (Two-tailed Student’s *T*-test).

Having demonstrated a possible inhibitory role for ERIC in E2F1-mediated apoptosis, we next investigated the function of ERIC in a chemotherapeutic agent-induced apoptosis of transformed cells. Administration of the chemotherapeutic drug etoposide to U2OS cells resulted in apoptotic cell death (Figure 5A inner panel) and a concomitant increase in ERIC RNA levels (Figure 5A). This damage-induced increase in ERIC RNA levels is most probably E2F1-independent since we detect a decrease in E2F1 protein levels after damage (Figure 5B lower panel), nevertheless it represents an upregulation of ERIC in response to a physiological stimulus. Silencing of ERIC, using two distinct siRNAs (Figure 5B), did not affect cell viability (Figure 5C left histograms) but when combined with etoposide it resulted in a significant augmentation of etoposide-induced apoptosis (Figure 5C, right histograms). These results were also evident in H1299 cells (Additional file 5: Figure S4). These results suggest that ERIC inhibits etoposide-induced apoptosis, thereby modulating the cellular response to chemotherapy.

## Discussion

As a master regulator for gene expression, E2F1 is able to directly or indirectly regulate numerous protein-coding and non-coding genes, especially microRNAs. For example, several groups have reported that miR-449a and miR-449b are induced by E2F [40,41]. Similarly, we have shown that expression of miR-15 and miR-16 is up-regulated by E2F1 [42]. The present study suggests that lncRNAs can also be subject to E2F1 regulation. Aberrant expression of lncRNAs is often associated with human diseases, in particular cancer [43].

In this study, we demonstrated that ERIC expression is regulated by E2Fs. Specifically, we show that activation of ectopic E2F1 or E2F3 result in elevated levels of ERIC RNA. The effect of E2F1 is direct and p53-independent. Moreover, we demonstrate that expression of this lncRNA is induced by E7, a viral protein that derepresses endogenous E2Fs. Furthermore, we show that ERIC expression is reduced in response to knock down of endogenous E2F1 or endogenous E2F3 and endogenous E2F1 binds ERIC’s promoter. Having established E2F regulation of ERIC, we also provide evidence that its RNA levels are elevated, in an E2f1-dependent manner, during resumption of growth following cell cycle arrest (Figure 3). Also, ERIC RNA levels are elevated in response to DNA damage (Figure 5A) [27]. Recent studies have identified lncRNAs whose expression is affected by cell cycle progression [3] or genotoxic stress [44]; however, regulation of their expression by specific transcription factors has not been studied in depth.

The expression of the lncRNAs H19 and ANRIL was shown to be regulated by E2F1 [45,46] and, to our knowledge, ERIC is the third lncRNA reported to be subject to E2F-regulation. Our RNA-Seq data suggest that more than a hundred lincRNAs are reproducibly regulated by E2F1; therefore, H19, ANRIL and ERIC most probably represent just the tip of the iceberg with regard to the full repertoire of E2F-regulated lncRNAs. Validating the regulation of additional lncRNAs by E2F as well as elucidating the role(s) such lncRNAs play in E2F1-mediated biological functions awaits future research. Clearly, further characterization of those potential lncRNAs will increase our understanding of how lncRNAs are integrated into the E2F-regulatory network.

The biological function(s) of lncRNAs are only now beginning to be elucidated; we show here that inhibition of ERIC expression augments E2F1-induced as well as etoposide-induced apoptosis (Figure 4, 5), suggesting that ERIC has an inhibitory effect on apoptosis in these settings.

The mode of action of ERIC remains to be determined. Most lncRNAs are nuclear [47] and many of them function in the regulation of gene expression. Our data, indicating that ERIC is largely nuclear (not shown), are in agreement with such a mechanism. Moreover, our DNA microarray data show that upon combined treatment of E2F1 activation and ERIC silencing in U2OS cells the expression of some E2F1-regulated apoptosis-related genes is significantly altered in a manner that is in agreement with enhancement of E2F1-induced apoptosis by ERIC silencing. However, the detailed effect of ERIC on gene expression awaits further studies.

With respect to the inhibitory effect of ERIC on E2F1-induced apoptosis, our data suggest the existence of a negative feedback loop: E2F1 activates expression of the lncRNA ERIC, which in turn, restricts the apoptotic function of E2F1.

This is not the first example of such a negative feedback loop regulating E2F activity, and in fact the RB/E2F pathway encompasses many such negative feedback loops. For example, we showed previously that E2F1 activates AKT via transcriptional regulation of the adaptor Gab2, and that this E2F1-dependent AKT activation serves to inhibit E2F1-mediated apoptosis [48]. Also, E2F1 and E2F3 were shown to transcriptionally up-regulate the expression of a number of miRNAs that can, in turn, inhibit cell proliferation [42,49].

We show here that ERIC is transcriptionally regulated by E2F1 and can mitigate E2F1 apoptotic activity. We propose that this lncRNA might fine-tune E2F1 activity, thereby preventing excessive E2F1 activity, which could harm normal cells. For example, its increase as arrested cells resume growth (Figure 3) may be one of the molecular mechanisms inhibiting E2F1-induced apoptosis at the G1/S transition. Of course, other mechanisms have been suggested to inhibit E2F1-induced apoptosis at the G1/S transition, for example regulation via the Akt pathway [50]. ERIC may be another layer in a multilayer regulatory pathway that prevents E2F1-induced apoptosis from occurring at the “wrong” time.

With respect to the inhibitory effect of ERIC on etoposide-induced apoptosis, our data demonstrate that ERIC RNA levels increase in response to DNA damage, and inhibition of ERIC expression augments damage-induced apoptosis. Thus, the anti-apoptotic function of ERIC is not limited to the artificial setting of ectopic E2F1 activation, as we showed it to play a role also in a more physiological setting, the cellular response to DNA damage by a chemotherapeutic agent. ERIC may have cancer-promoting effects, as well as conferring chemo-resistance, as it supports survival in the face of DNA damage. Thus, our data suggest that in the future, the level of ERIC RNA may serve as one of the parameters to predict patient response to DNA damaging chemotherapeutic drugs. Clearly, additional work is required to establish the predictive power of ERIC levels.

We and others have previously shown that ectopic expression of E2F1 synergizes with chemotherapeutic drugs in inducing apoptosis [51-53], however other studies showed that under some conditions, elevated E2F1 activity can lead to chemo-resistance [54]. The E2f1-induced upregulation of ERIC may represent one of several molecular mechanisms underlying such E2F1-mediated modulation of the response to chemotherapeutic drugs.

## Conclusions

This study reveals a novel regulatory loop, consisting of the transcription factor, E2F1, and a novel E2F1-regulated lncRNA XLOC 006942, which we have named ERIC. This loop is shown here to influence cell viability and thus, to control cell fate. Furthermore, our findings demonstrate that ERIC modulates the cellular response to chemotherapy. Given the key role of the E2F network in cancer biology, better understanding of the crosstalk between the players, such as between ERIC and E2F, should ultimately advance our understanding of cancer biology, and our ability to develop effective therapies.

## Materials and methods

### Cell culture

U2OS and SAOS-2 osteosarcoma cells were grown in Dulbecco’s modified Eagle’s medium supplemented with 5% fetal calf serum (FCS). Early passage WI38 human embryonic lung fibroblasts were grown in minimal essential medium supplemented with 10% fetal calf serum, 2 mM L-glutamine, 1 mM sodium pyruvate and non-essential amino acids. H1299 human lung adenocarcinoma cells were grown in RPMI 1640 medium supplemented with 5% fetal calf serum. Cells were maintained at 37°C in a humidified atmosphere containing 8% CO_2_. To induce activation of ER-E2F1, cells were treated with 100 nM 4-hydroxytamoxifen (OHT, Sigma) for the times indicated. Where indicated, cycloheximide (Sigma) was administered for 8 hr at 10 μg/ml. Etoposide (Sigma) was used at 50 μg/ml.

### Quantitative PCR (Real-Time RT- PCR)

Total RNA was extracted from the cells using the Tri Reagent method. Real-time quantitative PCR (qPCR) was done using SYBR Green PCR Master Mix (Applied Biosystems) with the following primer pairs:

GAPDH: 5′-CATGTTCCAATATGATTCCACC and 5′-GATGGGATTTCCATTGATGAC

XLOC 006942 (ERIC): 5′ -AGCCTGTGGCTACCTCCTTT and 5′ –CTTGCACCCATATGCAGACA, E2F3: 5′- CACCCTGGACCTCAAACTGT and 5′- AAGGCCACTAATTTTTCGAATATC.

All real-time reverse transcriptase PCR (RT-PCR) reactions were performed using the Applied Biosystems StepOnePlus Real-Time PCR Systems Machine. Results are presented as mean and SD for duplicate runs.

### Western blotting

Cells were lysed in lysis buffer [50 mmol/L Tris (Ph 7.5), 150 mmol/L NaCl, 1 mmol/L EDTA, 1% NP40] in the presence of protease inhibitor cocktail (Roche) and phosphatase inhibitor cocktails I and II (Sigma). Equal amounts of protein, as determined by the Bradford assay, were resolved by electrophoresis in a SDS 10% or 12.5% polyacrylamide gel and then transferred to a PVDF membrane (Millipore). The membrane was incubated with one of the following primary antibodies: anti-cleaved caspase-3 (Cell Signaling); anti-E2F1 (sc-251, Santa Cruz Biotechnology); anti-CCNE1 (sc-247, Santa Cruz Biotechnology); anti-E2F3 (sc-878, Santa Cruz Biotechnology); anti-actin (sc-1616r, Santa Cruz Biotechnology); anti-tubulin (T9026, Sigma); anti-GAPDH (sc-25778, Santa Cruz Biotechnology) or anti-P53 (sc-126, Santa Cruz Biotechnology). Binding of the primary antibody was detected using an enhanced chemiluminescence kit (ECL Amersham).

### Plasmids

The plasmids pBabe-neo-HA-ER-E2F1, pBabe-puro-HA, pBABE-puro-16E7 and pBABE-puro-E7-dl21-35 [55], pCDNA3.1(+), pCDNA3-E2F1, pCDNA3.1-HA-E2F1-E132 and pRETROSUPER-shp53.

### Transfection/infection procedures

To generate retroviruses, cells (2 × 106) of the packaging cell line 293 T were cotransfected with ecotropic packaging plasmid pSV-EMLV (10 μg), which provides packaging helper function, and the relevant plasmid (10 μg) using the calcium phosphate method in the presence of chloroquine (Sigma). After 8 hr, the transfection medium was replaced with fresh Dulbecco’s modified Eagle’s medium supplemented with 5% fetal calf serum. Subsequently, cell supernatants containing retroviruses were collected.

For infection, cells were incubated for 5 hr at 37°C in 4.5 mL of retroviral supernatant supplemented with polybrene (8 μg/mL, Sigma H9268). Then, 5.5 mL of medium was added and after a further 24 hr, the medium was replaced with fresh medium containing puromycin (2 μg/mL, Sigma P7130).

When transfecting U20S, SAOS-2 or H1299 cells with siRNA, Interferin transfection reagent (PolyPlus-transfection) was employed according to the manufacturer’s instructions. The siRNAs against ERIC (si#1: AAGCCAGCCTGTGGCTACCTCCTTT, si#2: CCCGTGGCATCGGCTGTCTGCATAT), SiE2F1(si#1- CUGAGGAGUUCAUCAGCCU si#2- CAGAGCAGAUGGUUAUGGU), siE2F3 (si#1- GCCUUAAAGACCAAACUGU, si#2- CAUAUCAAGAUAUUCGAAA) and a control sequence (siRNA universal negative control #1), were synthesized by Sigma-Aldrich. Experiments were performed 48 hours after transfection with siRNAs. When transfecting U20S cells with plasmids, polyjet transfection reagent (Signagen) was employed according to the manufacturer’s instructions.

### Chromatin immunoprecipitation

DNA–protein complexes were immunoprecipitated from U2OS cells using the ChIP (chromatin immunoprecipitation) assay kit (Upstate Biotechnology) according to the manufacturer’s protocol with the following antibodies: anti-E2F1 (sc-193; Santa Cruz Biotechnology) and anti IgG (111-035-144, Jackson Immunoresearch). Anti-IgG served as a control for nonspecific DNA binding. The precipitated DNA was subjected to RT- PCR analysis using specific primers corresponding to the human ERIC promoter (5′- GCCTCGCCAAACAGGTTTAC and 5′- ACTACAGAAACACGGAGGTCG) as well as primers for β-Actin that served as a negative control (5′- ACGCCAAAACTCTCCCTCCTCCTC and 5′- CATAAAAGGCAACTTTCGGAACGGC).

### Fluorescence-activated cell sorting analysis

Cells were trypsinized and then fixed by incubating in 70% ethanol at 4°C overnight. After fixation, cells were centrifuged for 4 min at 1500 rpm, and the pellet resuspended and incubated for 30 min at 4°C in 1 ml of PBS. Then, the cells were centrifuged again and resuspended in PBS containing 5 mg/ml propidium iodide and 50 μg/mL RNase A. After incubation for 20 min at room temperature, fluorescence intensity was analyzed using a Becton Dickinson flow cytometer.

### RNA Seq analysis

Six RNA samples (three from U2OS and three from H1299) were analyzed by Illumina Genome Analyzer IIx. Ribosomal RNA was removed from total RNA samples (using Ribo-Minus kit) to enrich mRNA concentration. Random primers were used to produce cDNA. Each RNA sample contained approximately 30 million reads with a read length of 36 nucleotides. The reads were aligned to the human genome (hg19) using bowtie (v0.12.8) [56] and TopHat (v1.2.0) [57]. For each sample, approximately 10 million reads were successfully aligned to the genome. Read counts per gene were calculated for each sample with htseqcount (http://www-huber.embl.de/users/anders/HTSeq). DESeq (v1.2.1) [58] was used to identify genes that were differentially expressed across the conditions.

## Competing interests

All the authors hereby declare that they do not have any competing interests with regard to the manuscript submitted here for review.

## Authors’ contributions

OF and TN- carried out the molecular biology studies, participated in the RNA Seq. analysis and drafted the manuscript. TD-performed the RNA Seq. analysis. GR and JJ designed and performed the RNA Seq. assay. DG- conceived of the study, and participated in its design and coordination and drafted the manuscript. All authors read and approved the final manuscript.

## Supplementary Material

Additional file 1: Table S1 LincRNAs that are expressed in at least one of the studied cell lines. Data collected from RNA sequencing analysis that was performed using U2OS and H1299 cells containing an ER-E2F1 expression vector. Table contains number of reads and fold induction results of lincRNAs levels after OHT treatment for 8 and 16 hours. Marked in yellow- 137 lincRNAs that were upregulated more than two fold at both time points in at least one of the cell lines after activation of E2F1. Inf (infinity)-represents a positive number divided by zero meaning, lincRNA is not expressed in basal state (before E2F1 induction). NA (not available) - represents division of zero by zero meaning, lincRNA is not expressed both before and after E2F1 activation. Click here for file

Additional file 2: Figure S1 E2F3 expression regulates ERIC RNA levels. **A)** U2OS cells containing conditionally active E2F3 were induced to activate E2F3 by addition of 4- hydroxyTamoxifen (OHT) for the times indicated. ERIC RNA levels were determined by Real-time RT-PCR and normalized to GAPDH. **B)** Upper panel- U2OS cells were transfected with either a nonspecific siRNA (siNS) or an siRNA directed against E2F3 (si#1 and si#2). RNA was extracted from the cells and E2F3 RNA levels determined by Real-time RT-PCR and normalized to GAPDH levels. Lower panel- Proteins were extracted from cells and western blot analysis performed using antibodies directed against E2F3 and GAPDH. **C)**. RNA extracted from cells described in B and ERIC RNA levels determined by Real-time RT-PCR and normalized to GAPDH levels. Click here for file

Additional file 3: Figure S2 Endogenous E2F1 regulates expression of ERIC in SAOS-2 cells. SAOS-2 cells were transfected with either a nonspecific siRNA (siNS) or an siRNA directed against E2F1 (siE2F1). Upper panel- RNA was extracted and ERIC RNA levels determined by Real-time RT-PCR and normalized to GAPDH levels. Lower panel- Proteins were extracted from cells and western blot analysis performed using antibodies directed against E2F1 and GAPDH. Orit-replace with a single si figure + change legend accordingly. Click here for file

Additional file 4: Figure S3 ERIC restricts E2F1-mediated apoptosis in H1299 cells. H1299 cells stably expressing ER-wild type E2F1 were transfected with either a nonspecific siRNA (siNS) or an siRNA directed against ERIC (si#1 or si#2). Cells were then left untreated or incubated with OHT (100 nM) for 20 hours. **A)** RNA was extracted, and ERIC RNA levels were determined by real-time RT-PCR and normalized to GAPDH levels. One representative experiment is shown. **B)** Proteins were extracted from the cells, and western blot analysis was performed using antibodies directed against cleaved caspase 3 and tubulin. Click here for file

Additional file 5: Figure S4 ERIC restricts DNA damage induced apoptosis in H1299 cells. **A)** H1299 cells were transfected with either a nonspecific siRNA (NS) or an siRNA directed against ERIC (si#1 or si#2). Then, cells were left untreated or incubated with etoposide (Etop) (150 μgr/ml) for 20 hours. Upper panel- RNA was extracted and ERIC RNA levels were determined by real-time RT-PCR and normalized to GAPDH levels. One representative experiment is shown. Lower panel- Proteins were extracted from the cells, and western blot analysis performed using antibodies directed against cleaved caspase 3 and actin. **B)** Cells were analyzed by FACS using propidium-iodide (PI) staining. One representative experiment is shown. Numbers represent percent of cells with a subG1 DNA content. Click here for file
