# Supplementary figures and images for: The long non-coding RNA ERIC is regulated by E2F and modulates the cellular response to DNA damage
Source: Mol Cancer. 2013 Oct 29;12:131. doi: 10.1186/1476-4598-12-131 (PMC4176120; doi:10.1186/1476-4598-12-131)

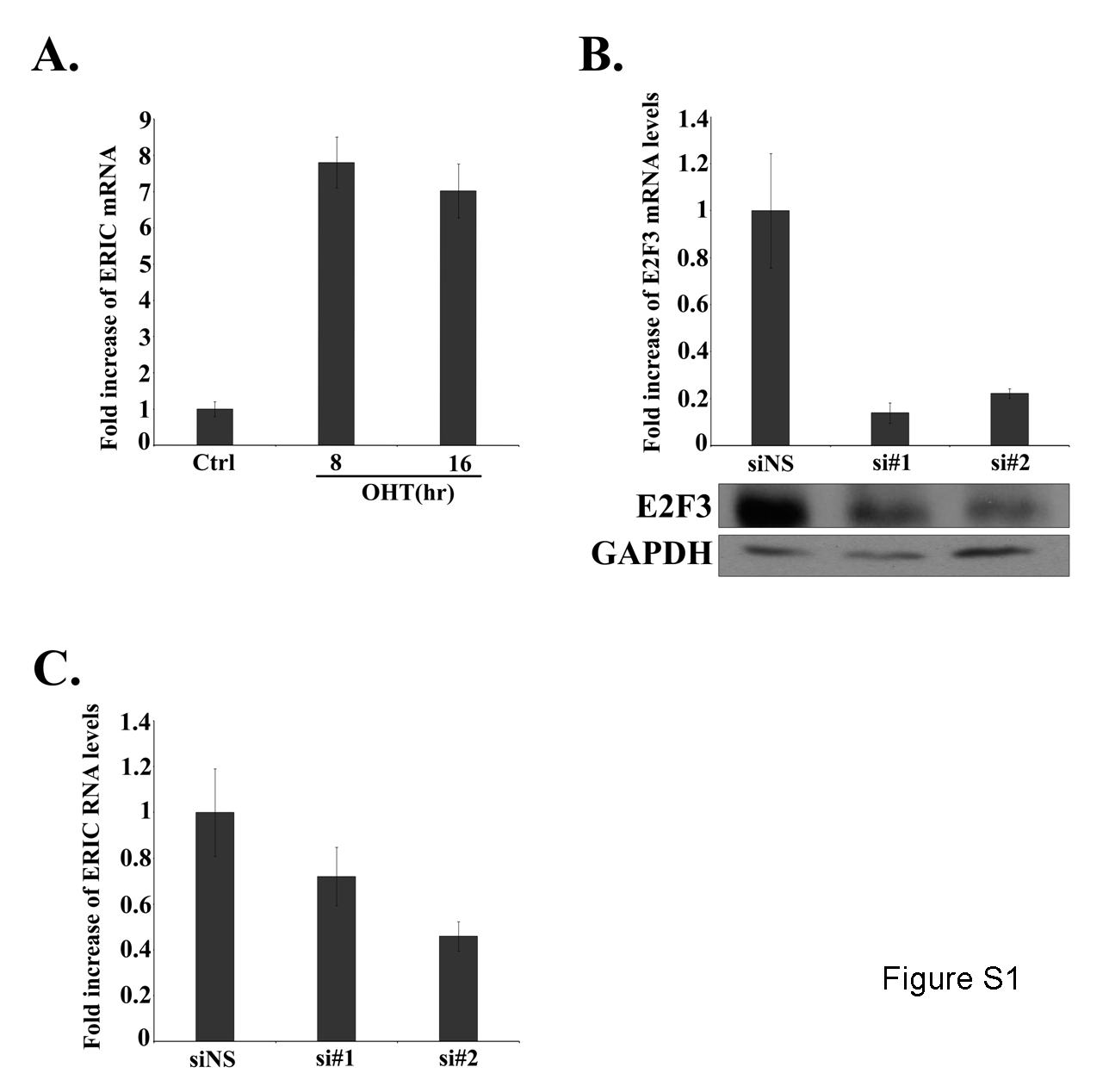

Supplement: Additional file 2: Figure S1 — E2F3 expression regulates ERIC RNA levels. A) U2OS cells containing conditionally active E2F3 were induced to activate E2F3 by addition of 4- hydroxyTamoxifen (OHT) for the times indicated. ERIC RNA levels were determined by Real-time RT-PCR and normalized to GAPDH. B) Upper panel- U2OS cells were transfected with either a nonspecific siRNA (siNS) or an siRNA directed against E2F3 (si#1 and si#2). RNA was extracted from the cells and E2F3 RNA levels determined by Real-time RT-PCR and normalized to GAPDH levels. Lower panel- Proteins were extracted from cells and western blot analysis performed using antibodies directed against E2F3 and GAPDH. C). RNA extracted from cells described in B and ERIC RNA levels determined by Real-time RT-PCR and normalized to GAPDH levels. [file 1476-4598-12-131-S2.tiff]

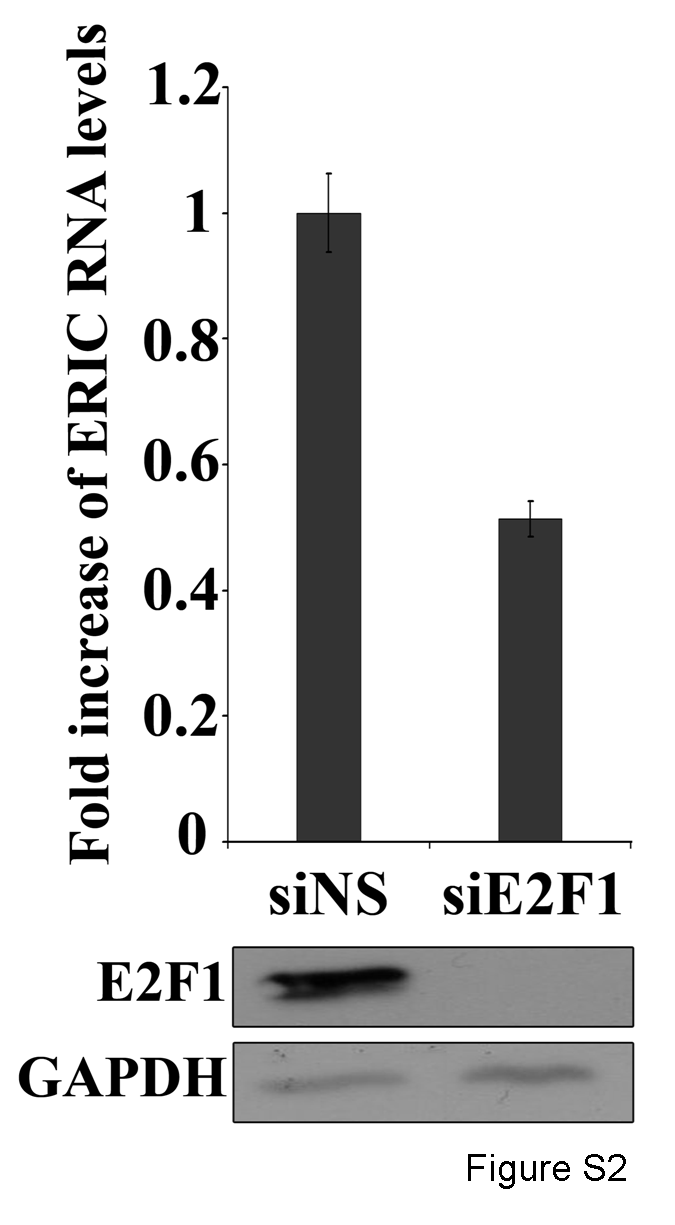

Supplement: Additional file 3: Figure S2 — Endogenous E2F1 regulates expression of ERIC in SAOS-2 cells. SAOS-2 cells were transfected with either a nonspecific siRNA (siNS) or an siRNA directed against E2F1 (siE2F1). Upper panel- RNA was extracted and ERIC RNA levels determined by Real-time RT-PCR and normalized to GAPDH levels. Lower panel- Proteins were extracted from cells and western blot analysis performed using antibodies directed against E2F1 and GAPDH. Orit-replace with a single si figure + change legend accordingly. [file 1476-4598-12-131-S3.tiff]

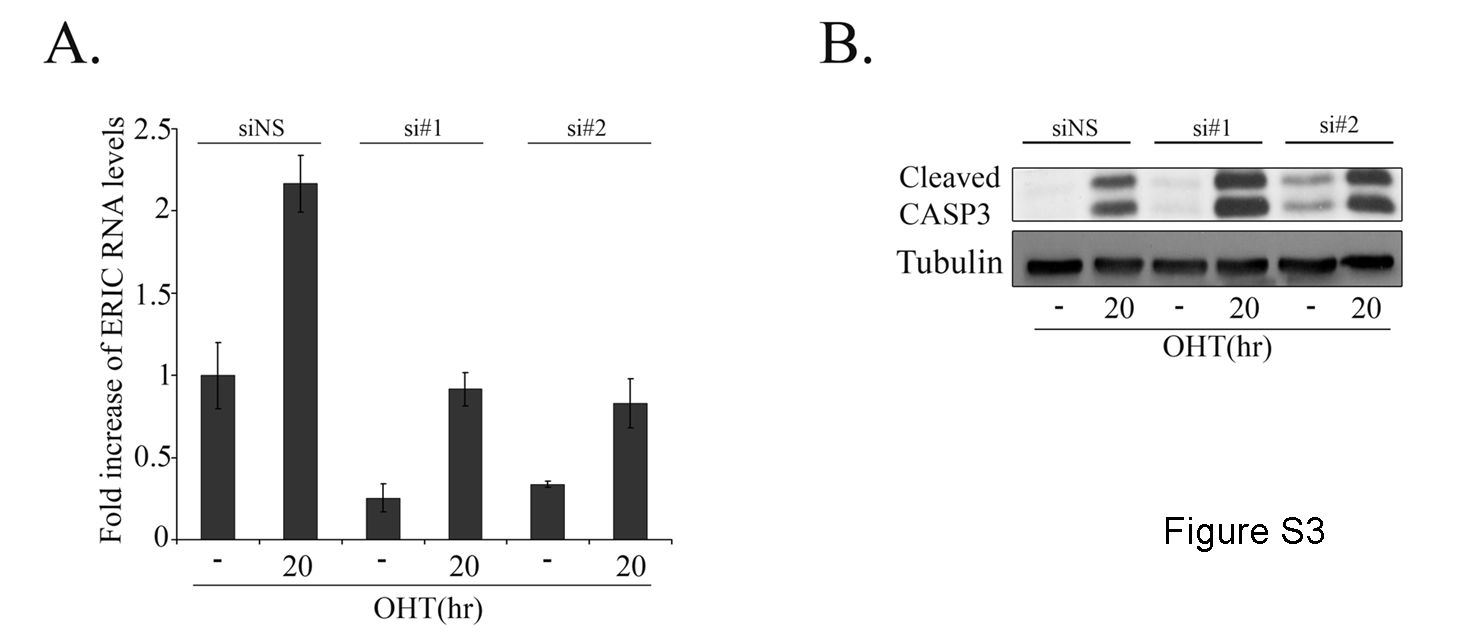

Supplement: Additional file 4: Figure S3 — ERIC restricts E2F1-mediated apoptosis in H1299 cells. H1299 cells stably expressing ER-wild type E2F1 were transfected with either a nonspecific siRNA (siNS) or an siRNA directed against ERIC (si#1 or si#2). Cells were then left untreated or incubated with OHT (100 nM) for 20 hours. A) RNA was extracted, and ERIC RNA levels were determined by real-time RT-PCR and normalized to GAPDH levels. One representative experiment is shown. B) Proteins were extracted from the cells, and western blot analysis was performed using antibodies directed against cleaved caspase 3 and tubulin. [file 1476-4598-12-131-S4.tiff]

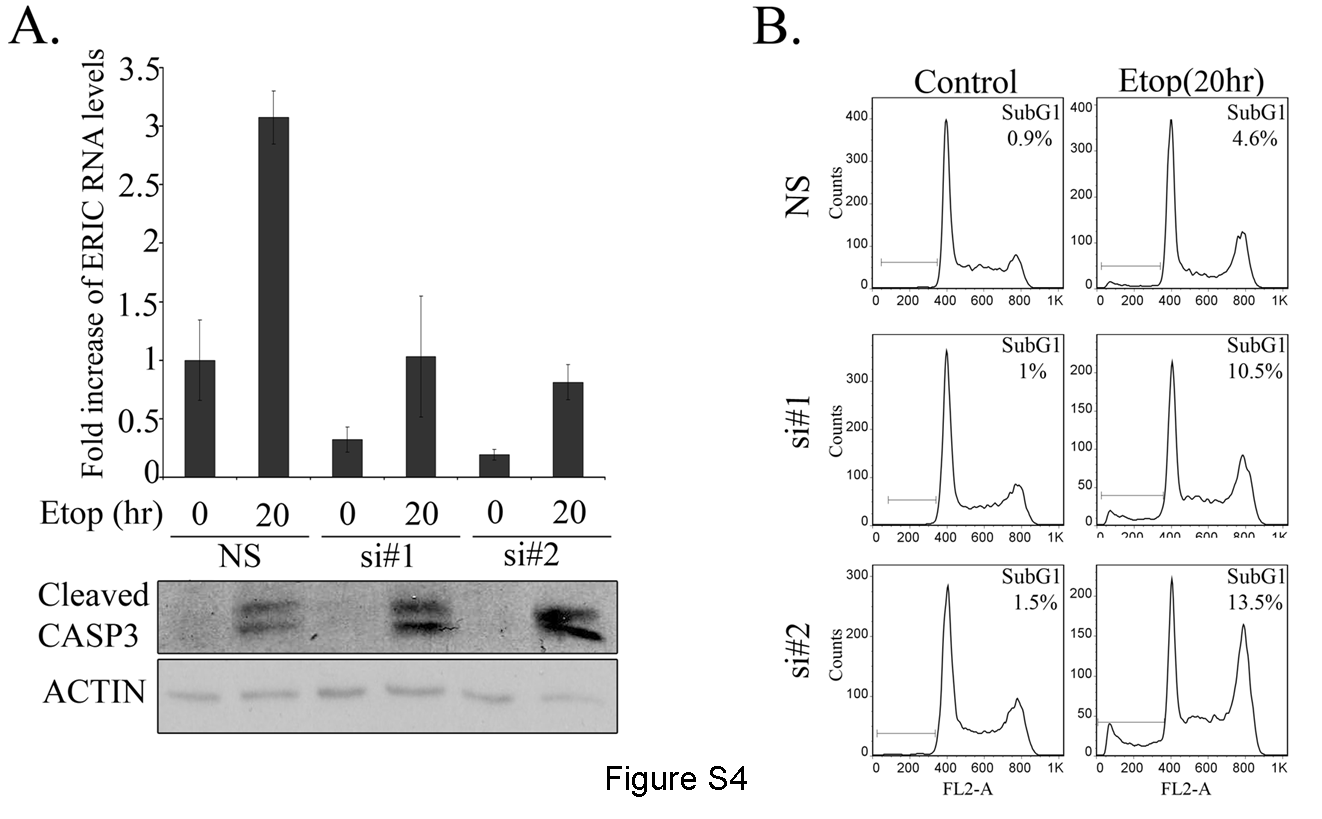

Supplement: Additional file 5: Figure S4 — ERIC restricts DNA damage induced apoptosis in H1299 cells. A) H1299 cells were transfected with either a nonspecific siRNA (NS) or an siRNA directed against ERIC (si#1 or si#2). Then, cells were left untreated or incubated with etoposide (Etop) (150 μgr/ml) for 20 hours. Upper panel- RNA was extracted and ERIC RNA levels were determined by real-time RT-PCR and normalized to GAPDH levels. One representative experiment is shown. Lower panel- Proteins were extracted from the cells, and western blot analysis performed using antibodies directed against cleaved caspase 3 and actin. B) Cells were analyzed by FACS using propidium-iodide (PI) staining. One representative experiment is shown. Numbers represent percent of cells with a subG1 DNA content. [file 1476-4598-12-131-S5.tiff]
